# Cardiorespiratory Coordination During Exercise in Adults With Down Syndrome

**DOI:** 10.3389/fphys.2021.704062

**Published:** 2021-09-08

**Authors:** Guillermo R. Oviedo, Sergi Garcia-Retortillo, María Carbó-Carreté, Myriam Guerra-Balic, Natàlia Balagué, Casimiro Javierre, Joan Guàrdia-Olmos

**Affiliations:** ^1^Faculty of Psychology, Education and Sport Science Blanquerna, University Ramon Llull, Barcelona, Spain; ^2^School of Health Science Blanquerna, University Ramon Llull, Barcelona, Spain; ^3^Keck Laboratory for Network Physiology, Department of Physics, Boston University, Boston, MA, United States; ^4^School of Health and Sport Sciences (EUSES), Universitat de Girona, Salt, Spain; ^5^Complex Systems in Sport Research Group, Institut Nacional d'Educació Física de Catalunya (INEFC) University of Barcelona, Barcelona, Spain; ^6^Serra Hunter Fellow, Department of Cognition, Development and Educational Psychology, Faculty of Psychology, University of Barcelona, Barcelona, Spain; ^7^Institute of Neuroscience, University of Barcelona, Barcelona, Spain; ^8^Department Physiological Sciences, University of Barcelona, Barcelona, Spain; ^9^Department of Social Psychology and Quantitative Psychology, Faculty of Psychology, University of Barcelona, Barcelona, Spain; ^10^Universitat de Barcelona Institute of Complex Systems, Barcelona, Spain

**Keywords:** Down syndrome, principal component analysis, cardiorespiratory fitness, blood pressure, network physiology of exercise, information entropy

## Abstract

**Introduction:** Down syndrome (DS) is a chromosomal disorder affecting simultaneously cardiovascular and respiratory systems. There is no research studying the coupling between these systems during cardiorespiratory exercise testing in a population with DS. Cardiorespiratory coordination (CRC), evaluated through principal component analysis (PCA), measures the covariation of cardiorespiratory variables during exercise.

**Objective:** To investigate and compare CRC in adults with and without DS during maximal cardiorespiratory exercise testing.

**Methods:** Fifteen adults with DS and 15 adults without disabilities performed a maximal cardiorespiratory exercise test on a treadmill. First, the slope, and afterward the velocity was increased regularly until participants reached exhaustion. The time series of six selected cardiorespiratory variables [ventilation per minute, an expired fraction of O_2_, the expired fraction of CO_2_, heart rate, systolic blood pressure (SBP), and diastolic blood pressure (DBP)] were extracted for the analysis. The number of principal components (PCs), the first PC eigenvalues (PC_1_), and the information entropy were computed for each group (non-DS and DS) and compared using a *t*-test or a Mann-Whitney U test.

**Results:** Two PCs in the non-DS group and three PCs in the DS group captured the variance of the studied cardiorespiratory variables. The formation of an additional PC in the DS group was the result of the shift of SBP and DBP from the PC_1_ cluster of variables. Eigenvalues of PC_1_ were higher in the non-DS (*U* = 30; *p* = 0.02; *d* = 1.47) than in the DS group, and the entropy measure was higher in the DS compared with the non-DS group (*U* = 37.5; *p* = 0.008; *d* = 0.70).

**Conclusion:** Adults with Down syndrome showed higher CRC dimensionality and a higher entropy measure than participants without disabilities. Both findings point toward a lower efficiency of the cardiorespiratory function during exercise in participants with DS. CRC appears as an alternative measure to investigate the cardiorespiratory function and its response to exercise in the DS population.

## Introduction

Down syndrome (DS), a relatively common chromosomal disorder, has always been part of the human condition. It exists in all regions of the world, and in addition to causing intellectual disability, it may cause varying effects on physical characteristics and an increased risk of developing medical conditions (World Health Organization, 2000). As of 2015, European DS population prevalence was estimated at 4.9 per 10,000 inhabitants and 6.7 per 10,000 in the United States (De Graaf et al., 2018). The literature comparing general physical health, as well as cardiorespiratory fitness between persons with DS and people without disabilities is extensive (Baynard et al., 2008; Franceschi et al., 2019; Gensous et al., 2020). However, there is no research focusing on the coupling between cardiovascular and respiratory systems and their response to exercise in the DS population. Therefore, the mechanisms underlying the coordinated activity between cardiovascular and respiratory systems to generate a particular function at the organism level in individuals with DS are still not fully understood.

Different studies have investigated the factors that may be influencing the impaired exercise response of persons with DS (Mendonca et al., 2010; Fernhall et al., 2013). Within these factors, researchers include musculoskeletal hypotonia (Antonarakis et al., 2020), lower walking economy and altered spatiotemporal gait variables (Agiovlasitis et al., 2009; Zago et al., 2020), altered autonomic function (Fernhall et al., 2013), reduced baroreceptors sensitivity (Agiovlasitis et al., 2010), attenuated adrenergic responsiveness during the process of exercise (Fernhall et al., 2009), and chronotropic incompetence (Guerra et al., 2003). While the most common cardiorespiratory parameters used to study the DS population offer useful information on diverse physiological systems separately, they do not provide sufficient information on the nature of the dynamic interactions between cardiovascular and respiratory systems and their common role as an integrated network (Bashan et al., 2012) to adjust the individual response to exercise requirements. Therefore, in the framework of the new field of Network Physiology (Bartsch et al., 2012, 2014, 2015; Ivanov and Bartsch, 2014; Rizzo et al., 2020) and, more specifically, of Network Physiology of Exercise (Balagué et al., 2020; Garcia-Retortillo et al., 2020, 2021), the use of non-linear models and time series analysis of coordinative variables are strongly recommended to identify interactions and principles of coordination and integration between diverse physiological systems (Schulz et al., 2013; Rivera et al., 2016, 2018; Barajas-Martínez et al., 2021). Several methodologies have been introduced in the past to investigate the complex interaction between cardiovascular and respiratory systems, from time and frequency domain measures (Horne, 2014) to more complex ones, such as phase synchronization and time-delay stability (Bartsch et al., 2012, 2014; Bartsch and Ivanov, 2014) or those related to information theory (Lucchini et al., 2018, 2020).

Several methodologies have been introduced in the past to investigate the complex interaction between cardiovascular and respiratory systems, from time and frequency domain measures (Horne, 2014) to more complex ones, such as those related to information theory (e.g., Bartsch et al., 2012; Lucchini et al., 2018, 2020). Such research approaches can track not only quantitative differences related to maximal and threshold physiological outcomes [e.g., heart rate (HR), VO_2_ max, ventilatory thresholds] but also qualitative changes in relation to the reallocation of resources under exercise-related constraints (Scholz and Schöner, 1999).

A recently proposed measure for testing the covariation of cardiorespiratory variables during maximal exercise testing (Balagué et al., 2016; Garcia-Retortillo et al., 2017, 2019a,b; Esquius et al., 2019; Zebrowska et al., 2020) is cardiorespiratory coordination (CRC). This is achieved through principal component analysis (PCA) conducted on the time series of several cardiorespiratory parameters. The PCA pinpoints and quantifies whether the increment and/or decrement of time patterns from different physiological processes are statistically correlated. In this way, the magnitude to which time patterns of physiological responses covary in time is reflected. The covariation of several (two or more) cardiorespiratory parameters shows the mutual information that they share. This common variance, in turn, enables time patterns of single cardiorespiratory outcomes to be represented through fewer principal components (PCs). The PCs are obtained in decreasing order of importance and reflect the highest possible fraction of the variability from the original dataset. Thus, the total number of PCs indicates the level of coordination among the initial cardiovascular and respiratory parameters. More concretely, a dimensionality reduction is indicative of the creation of new coordinative patterns (Hacken, 2010), therefore, the reduction in the quantity of PCs suggests an enhancement in the efficiency of CRC (Balagué et al., 2016). Entropy, in turn, is employed to calculate (i) the minimum information needed to determine the current state of a given system (Naudts, 2005) and (ii) the number of coordinative structures that are available for this system (Seely and Macklem, 2012). A greater quantity of accessible states reflects reduced covariation between the cardiovascular and respiratory parameters, changing more separately from another. Thus, measuring CRC through PCA and entropy could improve the understanding of the information yielded by the typically recorded performance and physiological parameters during cardiorespiratory exercise testing in the DS population, while showing the following advantages (refer to Balagué et al., 2016 for more details): (i) information on the level of covariation and co-relatedness among several cardiorespiratory variables, (ii) a decrease in the initial large dimensionality of the dataset making it easier to model, that is, from various cardiorespiratory variables to a few PCs, and (iii) information on the efficiency of specific training and research interventions to generate physiological adaptations.

Since DS is a chromosomal disorder affecting simultaneously cardiovascular and respiratory systems and given that such systems are interdependent and interact in a dynamic and non-linear way (Bartsch et al., 2012, 2014), we proposed in this study to use CRC, which has shown high responsiveness to various exercise and training contexts. To the best of our knowledge, CRC response to exercise has never been assessed in adults with DS, and it is not known whether it will provide further insight into the difference with respect to adults without DS in cardiorespiratory outcomes. Accordingly, the objective of this study was to investigate and compare CRC and cardiorespiratory variables between adults with and without DS during a maximal exercise test. We hypothesized that CRC and cardiorespiratory variables would differ between groups and that the impaired response to exercise of the DS group would be reflected in a lower degree of CRC and a higher entropy measure. This approach could offer new insights on how respiratory and cardiovascular systems coordinate their activation as a network, and how they synchronously integrate their function to generate a global response at the organism level. Therefore, understanding the dynamical organization and integration of respiratory and cardiovascular systems in adults with DS, as well as its response to exercise, would be of key relevance to develop new network-based biomarkers that can complement the information provided by the commonly utilized physiological variables to diagnose and track the evolution of the cardiorespiratory and hemodynamic parameters in adults with DS.

## Materials and Methods

### Participants

We conducted a power analysis utilizing G^*^Power 3.1 (Faul et al., 2007) to determine the sample size for this study. Previous studies of CRC during exercise (Balagué et al., 2016) have shown large effect sizes. Therefore, using an effect size of *d* = 1, α < 0.05, power (1—β) = 0.80, we estimated a sample size = 27. This cross-sectional study used a convenience sample of 15 adults (12 men and 3 women) with DS (*M* = 27.33; *SD* = 4.98 y.o.) and 15 adults (12 men and 3 women) without disabilities (non-DS; *M* = 27.01; *SD* = 4.60 y.o.). Participants were recruited from university campuses and occupational centers for adults with intellectual disabilities. All of them volunteered for this study. Volunteers between 18 and 35 years of age without disabilities and persons with DS of similar age were invited to a first meeting where the testing procedures, benefits, risks, and time required for the study were explained. During a second visit, all participants signed an informed consent form. Parents/legal guardians of participants with DS also signed the informed consent. All volunteers underwent a medical examination to discard any pathology and/or health problems that would not allow them to perform a maximal exercise test and received medical clearance to be part of this research. Inclusion criteria were as follows: (a) participants between 18 and 35 years old; (b) a normal 12-lead electrocardiogram at rest; (c) being able to walk without external aids; (d) willing to provide written informed consent, as well as the written consent of the parents/legal guardians of participants with DS. Exclusion criteria for both groups were to have the following: (a) cardiovascular diseases; (b) contraindications to exercise; (c) use of medications that may influence HR and/or exercise response; (d) inability to communicate orally; (e) inability to provide written informed consent; and (f) parents/legal guardians not willing to provide written informed consent.

The intellectual disability level of the participants was assessed by psychologists and categorized as borderline (one participant); mild (seven participants), and moderate (seven participants). We used the Kaufman Brief Test of Intelligence to assess the intelligence quotient of the participants (Kaufman and Kaufman, 1990).

This study was approved by the Institutional Review Board (CER URL 2017_2018_008) and complies with the principles of the Declaration of Helsinki (World Medical Association, 2013).

### Intervention and Procedure

To assess the peak aerobic capacity, all participants performed a cardiopulmonary exercise test on a treadmill (Quasar model, HP Cosmos sports & medical gmbh, Nussdorf-Traunstein, Germany). Participants walked at a constant speed (4 km/h), and the slope increased 2.5% every 2 min up to 12.5%. From that point on, the slope remained constant, and speed was increased 1.6 km/h every minute up to exhaustion. Exhaustion criteria were the following: HR and/or VO_2_ plateau, respiratory exchange ratio (RER) > 1.0, or when a participant could no longer continue. During the test, participants breathed through a two-way mask (Hans Rudolph, 2700, Kansas City, MO, USA) and gas exchange variables were determined breath-by-breath using an automated open-circuit system (Metasys TR-plus, Brainware SA, La Valette, France). Following the guidelines and recommendations of the manufacturer, gas and volume calibrations were performed prior to each test. Peak values were recorded as the highest value during the last 30 s of exercise.

**Figure 1:**
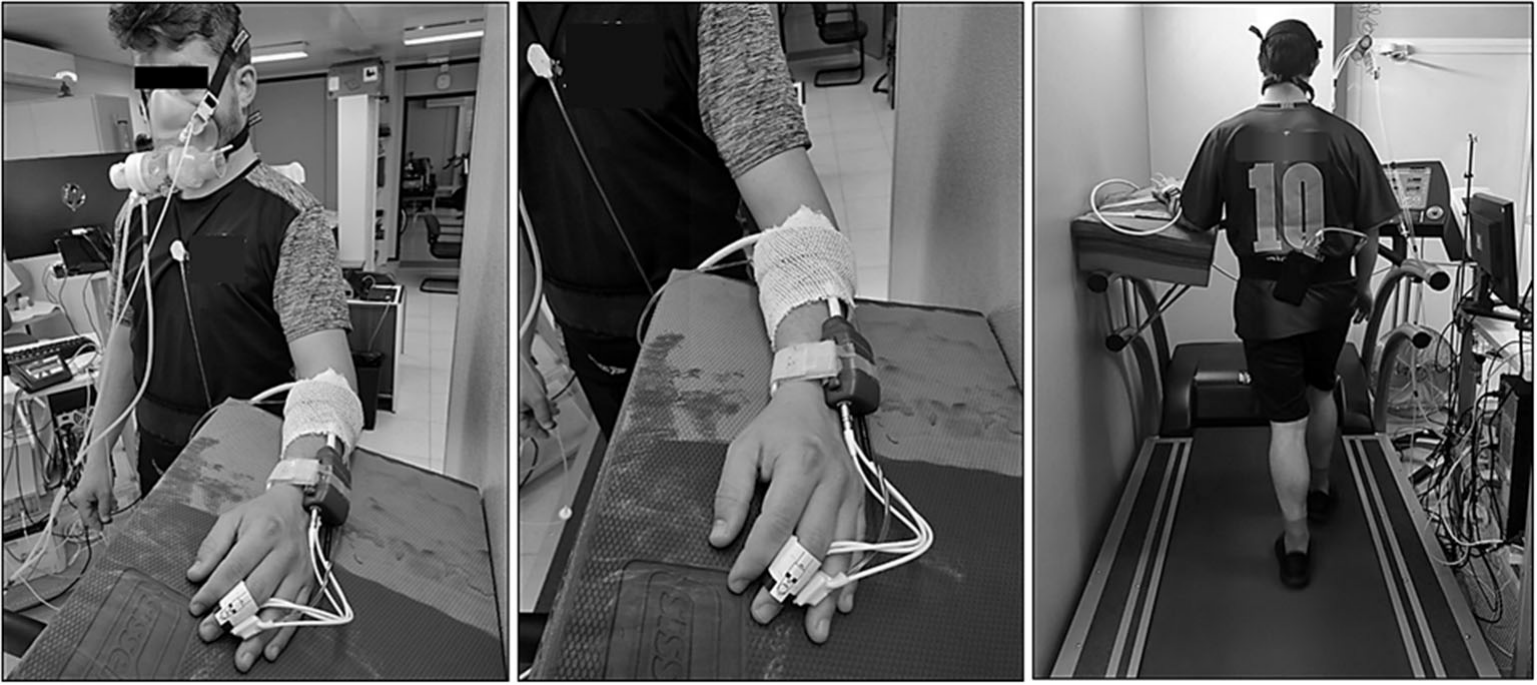
Example of a participant performing cardiopulmonary exercise test on the treadmill. In addition, it can be seen the placement of the finger cuff around the middle phalanx of the middle finger of the left hand.

The hemodynamic information obtained from the participants was continuously recorded by using finger cuff-based photoplethysmography (Nexfin, BMEYE Amsterdam, Netherlands). The finger sensor provided beat-to-beat blood pressure (BP) and determined systolic and diastolic BP (SBP and DBP). The finger cuff was placed around the middle phalanx of the middle finger of the left hand of the participants, and the left arm was relaxed and placed on a platform (refer to Figure 1). The technique used by the Nexfin device to estimate BP was described elsewhere (Martina et al., 2012; Garcia-Retortillo et al., 2017). Since it is useful for assessing acute changes in BP, we allowed the system to monitor continuously finger photoplethysmography (Eckert and Horstkotte, 2002).

Participants were monitored continuously *via* a 12-lead electrocardiogram (CardioScan v.4.0, DM Software, Stateline, Nevada, USA). All tests were conducted at an ambient temperature of 23°C and relative physical humidity of 48%, with variations of no more than 1°C in temperature and 10% in relative physical humidity. The tests were performed in the morning, and participants abstained from any moderate or vigorous exercise for at least 24 h before testing and refrained from alcohol and/or caffeine for at least 12 h before the assessments.

### Data Analysis

During the treadmill test, we registered the following cardiorespiratory variables: ventilation per minute (VE; L·min^−1^), oxygen uptake relative to body weight (VO_2_; ml·Kg^−1^·min^−1^), oxygen uptake (VO_2_; L·min^−1^), carbon dioxide production (VCO_2_; L·min^−1^), RER, expired fraction of O_2_ (FeO_2_; %), expired fraction of CO_2_ (FeCO_2_; %), SBP (mmHg), DBP (mmHg), HR (beat·min^−1^), ventilatory equivalent for O_2_ (VEqO_2_), ventilatory equivalent for CO_2_ (VEqCO_2_), and O_2_ pulse (ml·beat^−1^). The variables used for the specific PCA were obtained breath-by-breath. All data were tested for normality by using a Shapiro-Wilk test. The between-groups comparison (DS vs. non-DS) was performed using a *t*-test or, in the case of non-Gaussian distribution, using the Mann-Whitney U test. To analyze the CRC for each participant, we performed a PCA on the data series of the following selected cardiorespiratory variables: VE, FeO_2_, FeCO_2_, HR, SBP, and DBP. We excluded from the analysis VEqO_2_, VEqCO_2_, O_2_ pulse, RER, VO_2_, etc., due to their known deterministic mathematical relation (lineal combination) with the selected variables (Balagué et al., 2016). There is diverse evidence about the use of dimensionality reduction by PCA in small samples, which indicates certain robustness in the estimates of shared variance that Jolicoeur (1984) pointed out some time ago. In this sense, we must remember that the estimates in small samples should be more descriptive than inferential considerations, but appropriate to our objectives (Lang and Zou, 2020).

To analyze the suitability of the PCA implementation, we calculated Bartlett's test for sphericity and the Kaiser-Mayer-Olkin (KMO) test for all participants. We determined the number of PCs using the Kaiser-Gutmann criterion and thus considered PCs with eigenvalues λ ≥ 1.00 as significant (Jollife, 2002). Given that the first PC (PC_1_) always contains the highest proportion of the data variance, the PC_1_ eigenvalues were compared between non-DS and DS groups by means Mann-Whitney U test. Furthermore, the loadings of the six selected cardio-respiratory variables onto PC_1_, PC_2_, and PC_3_ were compared between groups using a Mann-Whitney U test.

Finally, to determine the degree of coordination between the cardiovascular and respiratory subsystems, the information entropy measure for both non-DS and DS groups was computed as previously indicated by Balagué et al. (2016): *H* = Sum [1/2 ln (EV) + 1/2 ln (3.14) + 1/2], where *H* is the entropy of the system and EV is the PC eigenvalue (Hacken, 2010). This sum includes all PC eigenvalues of each participant; for instance, for a subject with two PCs, the sum is repeated two times utilizing PC_1_ and PC_2_ eigenvalues. The information entropy measure between groups was compared by means of a Mann-Whitney U test. Alpha was set at *p* < 0.05 for all statistical tests. Effect size (Cohen's *d*) was calculated when possible to demonstrate the magnitude of standardized mean differences.

## Results

Table 1 depicts the descriptive characteristics of the participants. DS group had a similar weight but was shorter and had a higher BMI than the non-DS group (all *p* < 0.050).

**Table 1:** Characteristics of participants and peak cardiorespiratory values.

	**non-DS (** ***n*** **=** **15)**		**DS (** ***n*** **=** **15)**		***p*-value**
Characteristics					
Sex (male/female)	12/3		12/3		1.000
Age (years)	27.01	(4.60)	27.33	(4.98)	0.850
Height (m)	1.74	(0.07)	1.56	(0.06)	<0.001
Weight (kg)	70.08	(9.53)	66.58	(9.86)	0.331
BMI (kg/m^2^)	23.11	(1.99)	27.36	(4.29)	0.002
Peak cardiorespiratory values					
VE (L·min^−1^)	118.39	(23.69)	53.73	(14.49)	<0.001
VO_2_ (ml·Kg^−1^·min^−1^)	53.55	(10.42)	28.77	(6.67)	<0.001
VO_2_ (L·min^−1^)	3.76	(0.85)	1.90	(0.44)	<0.001
VCO_2_ (L·min^−1^)	4.42	(0.93)	2.12	(0.54)	<0.001
RER	1.18	(0.06)	1.12	(0.03)	0.002
FeO_2_ (%)	16.99	(0.44)	16.67	(0.39)	0.051
FeCO_2_ (%)	4.57	(0.35)	4.40	(0.39)	0.211
SBP (mmHg)	190.16	(28.79)	141.60	(22.66)	<0.001
DBP (mmHg)	104.12	(19.42)	85.56	(17.16)	0.010
HR (beat·min^−1^)	177	(13)	150	(13)	<0.001
VEqO_2_	31.82	(3.44)	28.17	(2.64)	0.003
VEqCO_2_	26.84	(1.79)	25.34	(2.32)	0.211
O_2_ pulse (ml·beat^−1^)	21.13	(4.38)	12.55	(2.46)	<0.001
Treadmill test duration (min)	17.60	(1.85)	12.50	(2.05)	<0.001

*Values are mean (SD). DS, Down syndrome; BMI, body mass index; VE, minute ventilation; VO_2_, oxygen uptake; VCO_2_, carbon dioxide production; RER, respiratory exchange ratio; FeO_2_, expired fraction of O_2_; FeCO_2_, expired fraction of CO_2_; SBP, systolic blood pressure; DBP, diastolic blood pressure; HR, heart rate; VEqO_2_, ventilatory equivalent for O_2_; VEqCO_2_, ventilatory equivalent for CO_2_; O_2_ pulse, oxygen pulse*.

Compared with the non-DS group, DS group reached lower physiological peak values: VE (*t* = 9.02; *p* < 0.001; *d* = 3.29); VO_2_ (*t* = 7.65; *p* < 0.001; *d* = 2.79); VCO_2_ (*t* = 8.82; *p* < 0.001; *d* = 3.22); RER (*t* = 3.41; *p* = 0.002; *d* = 1.24); SBP (*t* = 5.13; *p* < 0.001; *d* = 1.87), DBP (*t* = 2.77; *p* = 0.010; *d* = 1.01), HR (*t* = 5.47; *p* < 0.001; *d* = 1.99), VEqO_2_ (*t* = 3.26; *p* < 0.003; *d* = 1.19), and O_2_ pulse (*t* = 6.61; *p* < 0.001; *d* = 2.46). The duration of the tests performed by the non-disabled participants was longer than those performed by the DS participants (*t* = 7.97; *p* < 0.001; *d* = 2.91) (Table 1).

The Bartlett's sphericity test (*p* < 0.001) and the KMO index showed an acceptable sampling adequacy in both non-DS (*M* = 0.62; *SD* = 0.07) and DS groups (*M* = 0.57; *SD* = 0.07). Eigenvalues of PC_1_, representing the highest proportion of the data variance, were higher in the non-DS in contrast to the DS group (*U* = 30; *p* = 0.02; *d* = 1.47) (Table 2).

**Table 2:** PC_1_ eigenvalues and percentage of participants with two PCs (PC_2_) and three PCs (PC_3_) in non-Down syndrome and Down syndrome groups.

	**Non-Down Syndrome**				**Down Syndrome**			
	**Eigenvalues**		**% 2PCs**	**% 3PCs**	**Eigenvalues**		**% 2PCs**	**% 3PCs**
	**(PC_**1**_)**	**Entropy**			**(PC_**1**_)**	**Entropy**		
Mean	3.25	3.02	92.86	7.14	2.70	3.82	15.38	84.62
*SD*	0.36	0.31	–	–	0.39	0.43	–	–

*PC_1_, First Principal Component; % 2PC, percentage of participants with two principal components (PC); % 3PCs, percentage of participants with three principal components*.

**Figure 2:**
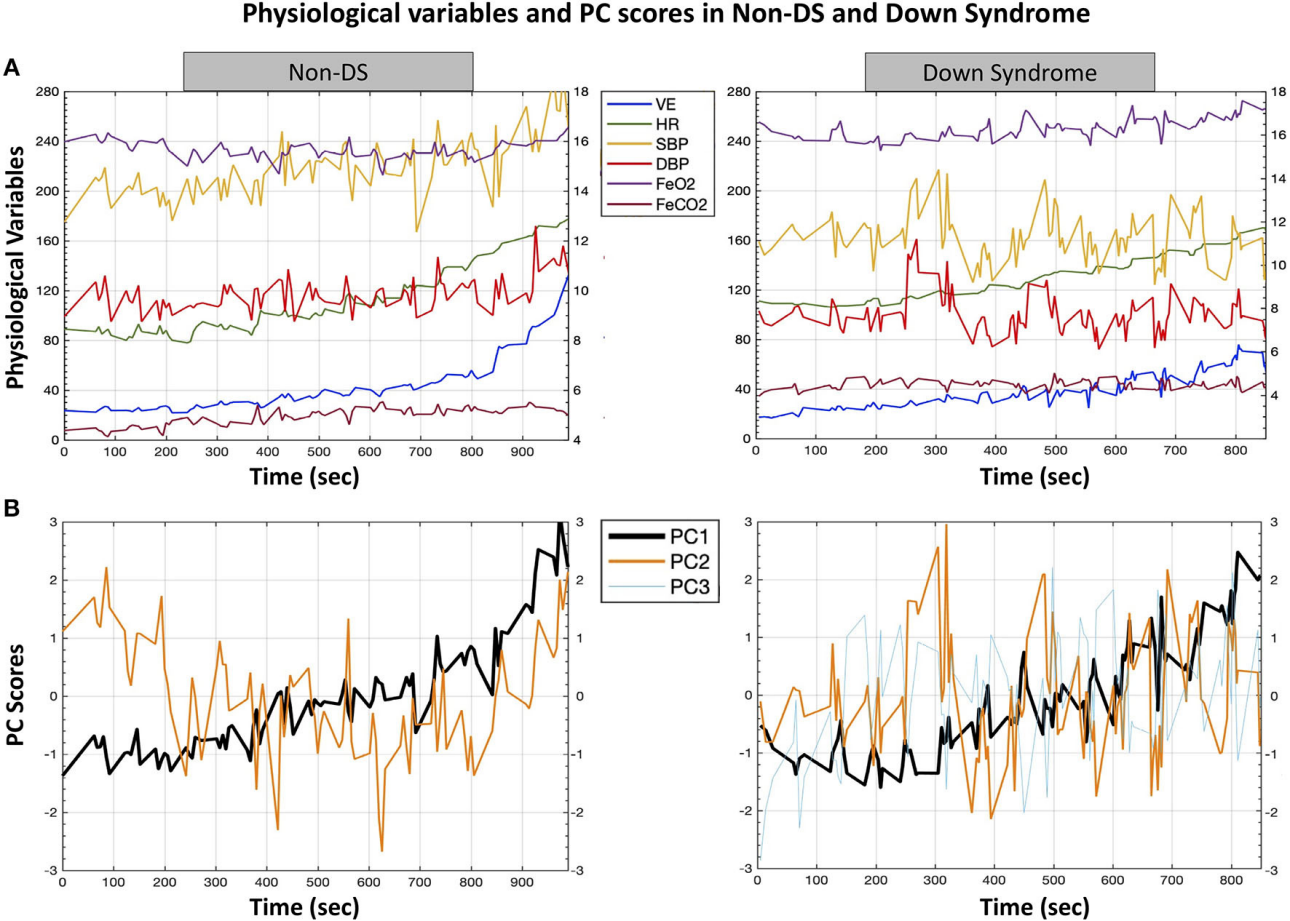
A typical example of the formation of time series of coordination variables, expressed by means of principal components from cardiorespiratory variables in non-DS and DS groups. This figure represents the higher number of PCs in the DS compared with the non-DS group (three PCs vs. two PCs, respectively), reflecting a lower degree of covariation of physiological variables, which can be linked to lower adaptive properties of the physiological network in response to exercise workloads. **(A)** Time series of the six selected cardiorespiratory variables during the cardiopulmonary exercise test. **(B)** Time series of the PC scores, with standardized z-values in the space spanned by PCs. The average trend was computed by the weighted least squares method. The variance of the six cardiorespiratory variables in the non-DS group was captured by two PCs: five variables (VE, FeCO_2_, HR, SBP, and DBP) were implicated in the formation of PC_1_, whereas PC_2_ was composed by a single variable (FeO_2_). However, the six selected cardiorespiratory variables could only be reduced to three PCs in the DS group: PC_1_ was formed by VE, FeCO_2_, and HR; PC_2_ by SBP and DBP, and PC_3_ was mainly saturated by FeO_2_. Note that the shift of SBP and DBP from the PC_1_ cluster of variables provoked the formation of an additional PC in the DS group. PC, principal component; VE, minute ventilation; HR, heart rate; SBP, systolic blood pressure; DBP, diastolic blood pressure; FeO_2_, expired fraction of oxygen; FeCO_2_, expired fraction of carbon dioxide; DS, Down syndrome.

As depicted in Figure 2, the variance of the six selected cardiorespiratory variables in the non-DS group was captured by two PCs: five variables (VE, FeCO_2_, HR, SBP, and DBP) were always implicated in forming PC_1_, whereas PC_2_ was composed of a single variable (FeO_2_). However, the six selected cardiorespiratory variables could only be reduced to three PCs in the DS group: PC_1_ was formed by VE, FeCO_2_, and HR; PC_2_ by SBP and DBP, and PC_3_ was mainly saturated by FeO_2_.

Accordingly, the loadings for SBP and DBP onto PC_1_ were significantly higher in the non-DS compared with the DS group: *U* = 46; *p* = 0.03; *d* = 0.78 and *U* = 40; *p* = 0.04; *d* = 0.77, respectively (Table 3).

**Table 3:** Average (SDs) for the loadings of the selected cardiorespiratory variables onto PC_1_.

	**Non-Down Syndrome**						**Down Syndrome**					
	**VE**	**FeO_**2**_**	**FeCO_**2**_**	**HR**	**SBP**	**DBP**	**VE**	**FeO_**2**_**	**FeCO_**2**_**	**HR**	**SBP**	**DBP**
Mean	0.87	0.05	0.61	0.90	0.43	0.35	0.84	0.28	0.49	0.88	0.06^*^	0.07^*^
*SD*	0.07	0.44	0.38	0.10	0.66	0.54	0.12	0.38	0.37	0.05	0.60	0.55

* *p < 0.05; VE, minute ventilation; FeO_2_, expired fraction of O_2_; FeCO_2_, expired fraction of CO_2_; HR, heart rate; SBP, systolic blood pressure; DBP, diastolic blood pressure*.

As for the loadings for PC_2_ (Table 4), while FeO_2_ loading was higher in the non-DS group (*U* = 45; *p* = 0.04; *d* = 0.50), loadings for SBP and DBP were higher in the DS group (*U* = 39.5; *p* = 0.001; *d* = 0.97 and *U* = 34; *p* = 0.005; *d* = 1.10). Note that it was the shift of SBP and DBP from the PC_1_ cluster of variables that provoked the formation of an additional PC in the DS group. Finally, reinforcing previous results, entropy measure was significantly higher in the DS compared with the non-DS group (*U* = 37.5; *p* = 0.008; *d* = 0.70) (Table 2).

**Table 4:** Average (SDs) for the loadings of the selected cardiorespiratory variables onto PC_2_.

	**Non-Down Syndrome**						**Down Syndrome**					
	**VE**	**FeO_**2**_**	**FeCO_**2**_**	**HR**	**SBP**	**DBP**	**VE**	**FeO_**2**_**	**FeCO_**2**_**	**HR**	**SBP**	**DBP**
Mean	0.25	0.50	−0.25	0.15	0.07	0.14	0.16	0.18^*^	−0.04	0.04	0.43^*^	0.54^*^
*SD*	0.29	0.66	0.58	0.27	0.30	0.37	0.32	0.63	0.48	0.33	0.43	0.36

* *p < 0.05; VE, minute ventilation; FeO_2_, expired fraction of O_2_; FeCO_2_, expired fraction of CO_2_; HR, heart rate; SBP, systolic blood pressure; DBP, diastolic blood pressure*.

## Discussions

To the best of our knowledge, this is the first study examining CRC in adults with DS and comparing their results with those of adults without disabilities of similar age and sex. As hypothesized, we found a higher number of PCs, reflecting a lower degree of CRC, and a higher entropy measure in the DS group in contrast to the non-DS group. In agreement with previous research, adults with DS showed lower values of cardiorespiratory fitness than their peers without disabilities (Baynard et al., 2008; Mendonca et al., 2011; Hilgenkamp et al., 2018).

There are several variables that may alter the cardiorespiratory function of people with DS (Fernhall et al., 2013). Among them, and derived from impaired autonomic function, are the reduced parasympathetic and sympathetic control and reduced baroreceptor sensitivity. These factors would lead to a lower cardiac response to exercise, lower BP, lower peripheral blood flow, and lower cardiac output, ultimately affecting cardiorespiratory fitness. In light of the current results, CRC has also been shown to be reduced, which allows us to explain and, in turn, is the consequence of previously mentioned findings. The higher number of PCs found in the DS group, compared with the non-DS group (three PCs vs. two PCs, respectively), reflects a lower degree of covariation among physiological variables, which can be linked to the lower adaptive properties of the physiological network in response to exercise workloads (Balagué et al., 2020). This may explain, together with the higher entropy measures observed in the DS group, the impaired efficiency of the cardiorespiratory system found in the DS population (Mendonca et al., 2010; Mendonca et al., 2018; Fernhall et al., 2013) and their commonly reduced fitness (Fernhall et al., 2013). More specifically, the higher entropy measure in the DS group is indicative of an increased number of coordinative states needed to specify physiological systems function during cardiorespiratory exercise testing, this reflects reduced covariation among the cardiovascular and respiratory variables, changing more separately from each other.

As for the non-DS group, our results support previous works investigating CRC in healthy individuals (Balagué et al., 2016; Garcia-Retortillo et al., 2017, 2019a,b; Esquius et al., 2019). The variance of the six cardiorespiratory variables during the cardiopulmonary exercise test was reduced to two PCs (Figure 2 and Tables 3, 4). The particular dynamics of FeO_2_, decreasing at onset as a result of initial hyperventilation (Skinner and McLellan, 1980), compared with the rest of the variables forming PC_1_ (with a clear increasing pattern during all the tests), was responsible for the formation of PC_2_ in the non-DS group. Regarding the DS group, the same FeO_2_ behavior was observed, leading to the formation of PC_3_ (Table 5). However, it is worth noting that the reduced CRC (i.e., the formation of an extra PC – PC_2_; Table 4) observed in the DS group was provoked by a change in SBP and DBP behavior (i.e., an erratic but correlated response of BP during the test; refer Figure 2), which subsequently led to a reduction in covariation between SBP and DBP, and the other cardiorespiratory variables forming PC_1_ (VE, FeO_2_, and FeCO_2_; Table 3). This response could be explained by the impaired autonomic function that may worsen with exercise intensity (Dipla et al., 2013; Hu et al., 2013). More specifically, individuals with DS show lower vagal withdrawal and diminished sympathetic responses to most sympatho-excitatory activities (Agiovlasitis et al., 2010), with smaller increments in BP during exercise (Bunsawat and Baynard, 2016). In response to sympatho-excitatory stimulation, DS individuals can preserve central pressure. However, a reduction in wave reflection was found during sympathetic stimulation, which could be indicative of a limited vasoconstriction capacity in the periphery (Hilgenkamp et al., 2019).

**Table 5:** Average (SDs) for the loadings of the selected cardiorespiratory variables onto PC_3_.

	**Non-Down Syndrome**						**Down Syndrome**					
	**VE**	**FeO_**2**_**	**FeCO_**2**_**	**HR**	**SBP**	**DBP**	**VE**	**FeO_**2**_**	**FeCO_**2**_**	**HR**	**SBP**	**DBP**
Mean	–	–	–	–	–	–	0.04	0.32	0.10	0.07	0.18	0.24
*SD*	–	–	–	–	–	–	0.19	0.56	0.63	0.19	0.36	0.39

*VE, minute ventilation; FeO_2_, expired fraction of O2; FeCO_2_, expired fraction of CO_2_; HR, heart rate; SBP, systolic blood pressure; DBP, diastolic blood pressure*.

Exercise challenges the adaptive capacity of physiological networks, providing relevant information about their coordinative properties. Therefore, CRC could be a privileged tool for testing the adaptability of the cardiorespiratory function, which may be altered under different fitness and health states. The response of the network to different exercise intensity perturbations may be better captured by coordinative variables (such as CRC or psychophysiological parameters) than through maximal cardiorespiratory variables (Balagué et al., 2014, 2020; Slapsinskaite et al., 2016).

The current results allow us to hypothesize that CRC, evaluated through a PCA of cardiorespiratory variables recorded during progressive exercise tests, may be an alternative assessment system of cardiorespiratory function in the DS population. It can help to increase the sensitivity of commonly registered fitness biomarkers (e.g., VO_2_ peak) and be more responsive to changes produced by exercise programs and training interventions. Previous results have demonstrated the higher responsiveness of CRC with respect to maximal performance and physiological variables after distinct training programs (Balagué et al., 2016; Garcia-Retortillo et al., 2019a), and under different fatigue states (Garcia-Retortillo et al., 2017), and nutritional interventions (Esquius et al., 2019). As individuals with DS and a low-fitness level population, in general, may have more difficulties in performing maximal tests, the assessment of CRC might be more advantageous since it does not require reaching maximal and peak performance values.

The findings of this work need to be discussed in light of some methodological limitations. In this study, the BP data obtained through a non-invasive finger cuff (refer to Methods section) may have been affected by some potential artifacts due to the test protocol changes on the treadmill gradient and speed. Such changes could produce a possible balance loss of participants who, in turn, could apply some isometric force with their hand affecting the data recorded by the finger cuff. Nevertheless, during the entire test on the treadmill, special care and control were taken to ensure that this did not happen. According to current results, motor coordination problems of the DS population can be extended to CRC. It has to be considered that motor coordination depends on both psychological and biological issues, therefore, in future studies, it may be of interest to take into account psychological variables that would give more power to the net. Finally, it is important to recognize that the interpretation of our findings should be treated with caution due to the reduced sample of participants with DS included in this study. Further research is needed to identify the mechanisms underlying the lower CRC levels as well as the potential relationship with the impaired autonomic function typically observed in DS patients. Finally, research is also warranted for analyzing in more detail the effects of different anthropometric variables, such as weight, height, BMI, percentages of fat mass, and fat-free mass on the CRC of people with DS.

In conclusion, adults with DS show lower CRC and higher entropy in contrast to adults without a disability. Our results revealed that the reduced CRC observed in the DS group is because the SBP and DBP of participants with DS behave differently than the BP of their peers without DS. We hypothesized that the difference in BP response during the treadmill test may be linked to an impaired autonomic regulation in the DS participants (Figueroa et al., 2005; Fernhall et al., 2013). However, this should be further investigated empirically. The CRC evaluation appears as an alternative measure for investigating the cardiorespiratory function and its response to exercise and training in the DS population, alongside traditional measures of aerobic fitness.

## Data Availability Statement

The raw data supporting the conclusions of this article will be made available by the authors, without undue reservation.

## Ethics Statement

The studies involving human participants were reviewed and approved by Comité de Ética e Investigación de la Universidad Ramon Llull. The patients/participants provided their written informed consent to participate in this study.

## Author Contributions

GO, SG-R, and MC-C conceived the manuscript and jointly drafted the content. SG-R and NB conceived the approach to data analysis. CJ and GO worked on the acquisition and analysis of the data. MG-B, CJ, NB, and JG-O edited and revised the manuscript. All authors approved the final version of the manuscript and agreed to be accountable for all aspects of the work.

## Funding

This study was partially supported by the Spanish Ministry of Economy, Industry, and Competitiveness (I + D + i Ref: DEP2017–86862-C2–1-R); by the Ministry of Science, Innovation and Universities State Research Agency (Ref: PGC2018-095829-B-I00), and by the Secretaria d'Universitats i Recerca del Departament d'Empresa i Coneixement de la Generalitat de Catalunya i la Universitat Ramon Llull (Ref: 2021-URL-Proj-042). The funders had not any role in the study design, data collection and analysis, decision to publish, or preparation of the manuscript.

## Conflict of Interest

The authors declare that the research was conducted in the absence of any commercial or financial relationships that could be construed as a potential conflict of interest.

## Publisher's Note

All claims expressed in this article are solely those of the authors and do not necessarily represent those of their affiliated organizations, or those of the publisher, the editors and the reviewers. Any product that may be evaluated in this article, or claim that may be made by its manufacturer, is not guaranteed or endorsed by the publisher.

## References

[B1] Agiovlasitis S. Collier S. R. Baynard T. Echols G. H. Goulopoulou S. Figueroa A.. (2010). Autonomic response to upright tilt in people with and without Down syndrome. Res. Dev. Disabil. 31, 857–863. 10.1016/j.ridd.2010.03.002 20307953

[B2] Agiovlasitis S. McCubbin J. Yun J. Pavol M. Widrick J. (2009). Economy and preferred speed of walking in adults with and without Down syndrome. Adapt. Phys. Act. Q. 26, 118–130. 10.1123/apaq.26.2.118 19478345

[B3] Antonarakis S. E. Skotko B. G. Rafii M. S. Strydom A. Pape S. E. Bianchi D. W.. (2020). Down syndrome. Nat. Rev. Dis. Prim. 6:2020. 10.1038/s41572-019-0143-7 32029743 PMC8428796

[B4] Balagué N. Aragonés D. Hristovski R. García-Retortillo S. Tenenbaum G. (2014). Attention focus emerges spontaneously during progressive and maximal exercise. Rev. Psicol. del Deport. 23, 57–63.

[B5] Balagué N. González J. Javierre C. Hristovski R. Aragonés D. Álamo J.. (2016). Cardiorespiratory coordination after training and detraining. A principal component analysis approach. Front. Physiol. 7:35. 10.3389/fphys.2016.00035 26903884 PMC4751338

[B6] Balagué N. Hristovski R. Almarcha M. Garcia-Retortillo S. Ivanov P. C. (2020). Network physiology of exercise: vision and perspectives. Front. Physiol. 11:611550. 10.3389/fphys.2020.611550 33362584 PMC7759565

[B7] Barajas-Martínez A. Tello-Santoyo g. Berumen-Cano P. Robles-Cabrera A. López-Rivera J. A. Fossion R.. (2021). Cardio-respiratory variability of healthy young subjects, in AIP Conference Proceedings, Vol. 2348 (AIP Publishing LLC).

[B8] Bartsch R. P. Ivanov P. C. (2014). Coexisting forms of coupling and phase-transitions in physiological networks in Communications in Computer and Information Science. Vol. 438, eds Mladenov V. M. Ivanov P. C. (Cham: Springer), 270–287.

[B9] Bartsch R. P. Liu K. K. Ma Q. D. Ivanov P. C. (2014). Three independent forms of cardio-respiratory coupling: transitions across sleep stages. Comput. Cardiol. 41, 781–784. 25664348 PMC4319215

[B10] Bartsch R. P. Liu K. K. L. Bashan A. Ivanov P. C. (2015). Network physiology: how organ systems dynamically interact. PLoS ONE 10:e0142143. 10.1371/journal.pone.0142143 26555073 PMC4640580

[B11] Bartsch R. P. Schumann A. Y. Kantelhardt J. W. Penzel T. Ivanov P. C. (2012). Phase transitions in physiologic coupling. Proc. Natl. Acad. Sci. U.S.A. 109, 10181–10186. 10.1073/pnas.1204568109 22691492 PMC3387128

[B12] Bashan A. Bartsch R. P. Kantelhardt J. W. Havlin S. Ivanov P. C. (2012). Network physiology reveals relations between network topology and physiological function. Nat. Commun. 3:702. 10.1038/ncomms1705 22426223 PMC3518900

[B13] Baynard T. Pitetti K. H. Guerra M. Unnithan V. B. Fernahall B. (2008). Age-related changes in aerobic capacity in individuals with mental retardation: a 20-yr review. Med. Sci. Sports Exerc. 40, 1984–1989. 10.1249/MSS.0b013e31817f19a1 18845971

[B14] Bunsawat K. Baynard T. (2016). Cardiac autonomic modulation and blood pressure responses to isometric handgrip and submaximal cycling exercise in individuals with down syndrome. Clin. Auton. Res. 26, 253–260. 10.1007/s10286-016-0361-y 27165540

[B15] De Graaf G. Buckley F. Skotko B. G. (2018). Birth and population prevalence for Down syndrome in European countries, in World Down Syndrome Congress (Glasgow).

[B16] Dipla K. Zafeiridis A. Papadopoulos S. Koskolou M. Geladas N. Vrabas I. S. (2013). Reduced metaboreflex control of blood pressure during exercise in individuals with intellectual disability: a possible contributor to exercise intolerance. Res. Dev. Disabil. 34, 335–343. 10.1016/j.ridd.2012.08.020 23000635

[B17] Eckert S. Horstkotte D. (2002). Comparison of Portapres non-invasive blood pressure measurement in the finger with intra-aortic pressure measurement during incremental bicycle exercise. Blood Press. Monit. 7, 179–183. 10.1097/00126097-200206000-00006 12131075

[B18] Esquius L. Garcia-Retortillo S. Balagué N. Hristovski R. Javierre C. (2019). Physiological- and performance-related effects of acute olive oil supplementation at moderate exercise intensity. J. Int. Soc. Sports Nutr. 16:12. 10.1186/s12970-019-0279-6 30823922 PMC6397506

[B19] Faul F. Erdfelder E. Lang A. G. Buchner A. (2007). G^*^Power 3: a flexible statistical power analysis program for the social, behavioral, and biomedical sciences. Behav. Res. Methods 39, 175–191. 10.3758/BF03193146 17695343

[B20] Fernhall B. Baynard T. Collier S. R. Figueroa A. Goulopoulou S. Kamimori G. H.. (2009). Catecholamine response to maximal exercise in persons with Down syndrome. Am. J. Cardiol. 103, 724–726. 10.1016/j.amjcard.2008.10.036 19231341

[B21] Fernhall B. Mendonca G. V. Baynard T. (2013). Reduced work capacity in individuals with down syndrome: a consequence of autonomic dysfunction? Exerc. Sport Sci. Rev. 41, 138–147. 10.1097/JES.0b013e318292f408 23558694

[B22] Figueroa A. Collier S. R. Baynard T. Giannopoulou I. Goulopoulou S. Fernhall B. (2005). Impaired vagal modulation of heart rate in individuals with Down syndrome. Clin. Auton. Res. 15, 45–50. 10.1007/s10286-005-0235-1 15768202

[B23] Franceschi C. Garagnani P. Gensous N. Bacalini M. G. Conte M. Salvioli S. (2019). Accelerated bio-cognitive aging in Down syndrome: state of the art and possible deceleration strategies. Aging Cell 18:e12903. 10.1111/acel.12903 30768754 PMC6516152

[B24] Garcia-Retortillo S. Gacto M. O'Leary T. J. Noon M. Hristovski R. Balagué N.. (2019a). Cardiorespiratory coordination reveals training-specific physiological adaptations. Eur. J. Appl. Physiol. 119, 1701–1709. 10.1007/s00421-019-04160-3 31187282

[B25] Garcia-Retortillo S. Javierre C. Hristovski R. Ventura J. L. Balagué N. (2017). Cardiorespiratory coordination in repeated maximal exercise. Front. Physiol. 8:387. 10.3389/fphys.2017.00387 28638349 PMC5461287

[B26] Garcia-Retortillo S. Javierre C. Hristovski R. Ventura J. L. Balagué N. (2019b). Principal component analysis as a novel approach for cardiorespiratory exercise testing evaluation. Physiol. Meas. 40:084002. 10.1088/1361-6579/ab2ca0 31239421

[B27] Garcia-Retortillo S. Rizzo R. Ivanov P. C. (2021). Spectral dynamics of muscle fiber activation in response to exercise and acute fatigue, in IEEE EMBS International Conference on Biomedical and Health Informatics (BHI) (Athens: IEEE), 1–4. 10.1109/BHI50953.2021.9508556

[B28] Garcia-Retortillo S. Rizzo R. Wang J. W. J. L. Sitges C. Ivanov P. C. (2020). Universal spectral profile and dynamic evolution of muscle activation: a hallmark of muscle type and physiological state. J. Appl. Physiol. 129, 419–441. 10.1152/japplphysiol.00385.2020 32673157 PMC7517426

[B29] Gensous N. Bacalini M. G. Franceschi C. Garagnani P. (2020). Down syndrome, accelerated aging and immunosenescence. Semin. Immunopathol. 42, 635–645. 10.1007/s00281-020-00804-1 32705346 PMC7666319

[B30] Guerra M. Llorens N. Fernhall B. (2003). Chronotropic incompetence in persons with down syndrome. Arch. Phys. Med. Rehabil. 84, 1604–1608. 10.1053/S0003-9993(03)00342-3 14639558

[B31] Hacken H. (2010). Information and Self-Organization. A Macroscopic Approach to Complex Systems, 3rd Edn. New York, NY: Springer, 69–152.

[B32] Hilgenkamp T. I. M. Schroeder E. C. Wee S. O. Grigoriadis G. Rosenberg A. J. Baynard T.. (2019). Altered central hemodynamics in individuals with Down syndrome. Artery Res. 25, 107–112. 10.2991/artres.k.191204.001

[B33] Hilgenkamp T. I. M. Wee S. O. Schroeder E. C. Baynard T. Fernhall B. (2018). Peripheral blood flow regulation in response to sympathetic stimulation in individuals with down syndrome. Artery Res. 24, 16–21. 10.1016/j.artres.2018.10.001 31105801 PMC6519939

[B34] Horne R. S. C. (2014). Cardio-respiratory control during sleep in infancy. Paediatr. Respir. Rev. 15, 163–169. 10.1016/j.prrv.2013.02.012 23523390

[B35] Hu M. Yan H. Ranadive S. M. Agiovlasitis S. Fahs C. A. Atiq M.. (2013). Arterial stiffness response to exercise in persons with and without Down syndrome. Res. Dev. Disabil. 34, 3139–3147. 10.1016/j.ridd.2013.06.041 23883823

[B36] Ivanov P. C. Bartsch R. P. (2014). Network physiology: mapping interactions between networks of physiologic networks, in Networks of Networks: The Last Frontier of Complexity. Understanding Complex Systems, eds D'Agostino G. Scala A. (Ithaca, NY: Springer), 203–222.

[B37] Jolicoeur P. (1984). Principal components, factor analysis, and multivariate allometry: a small-sample direction test. Biometrics 40, 685–690. 10.2307/2530911

[B38] Jollife I. T. (2002). Principal Component Analysis, 2nd Edn. New York, NY: Springer-Verlag.

[B39] Kaufman A. S. Kaufman N. L. (1990). Kaufman Brief Intelligence Test. Bloomington, MN: Pearson, Inc.

[B40] Lang W. Zou H. (2020). A simple method to improve principal components regression. Stat 9:e288. 10.1002/sta4.288

[B41] Lucchini M. Pini N. Burtchen N. Signorini M. G. Fifer W. P. (2020). Transfer entropy modeling of newborn cardiorespiratory regulation. Front. Physiol. 11:1095. 10.3389/fphys.2020.01095 32973570 PMC7481456

[B42] Lucchini M. Pini N. Fifer W. P. Burtchen N. Signorini M. G. (2018). Characterization of cardiorespiratory phase synchronization and directionality in late premature and full term infants. Physiol. Meas. 39:064001. 10.1088/1361-6579/aac553 29767630 PMC6063316

[B43] Martina J. R. Westerhof B. E. van Goudoever J. de Beaumont E. M. F. H. Truijen J. Kim Y.-S.. (2012). Noninvasive continuous arterial blood pressure monitoring with Nexfin®. Anesthesiology 116, 1092–1103. 10.1097/ALN.0b013e31824f94ed 22415387

[B44] Mendonca G. Pereira F. Fernhall B. (2010). Reduced exercise capacity in persons with Down syndrome: cause, effect, and management. Ther. Clin. Risk Manag. 6, 601–610. 10.2147/TCRM.S10235 21206759 PMC3012449

[B45] Mendonca G. Pereira F. Fernhall B. (2011). Effects of combined aerobic and resistance exercise training in adults with and without Down syndrome. Arch. Phys. Med. Rehabil. 92, 37–45. 10.1016/j.apmr.2010.09.015 21187203

[B46] Naudts J. (2005). Boltzmann entrogpy and the microcanonical ensemble. EPL 69, 719–724. 10.1209/epl/i2004-10413-1

[B47] Rivera A. L. Estañol B. Robles-Cabrera A. Toledo-Roy J. C. Fossion R. Frank A. (2018). Looking for biomarkers in physiological time series, in Quantitative Models for Microscopic to Macroscopic Biological Macromolecules and Tissues (Cham: Springer), 111–131.

[B48] Rivera A. L. Estanol B. Senties-Madrid H. Fossion R. Toledo-Roy J. C. Mendoza-Temis J.. (2016). Heart rate and systolic blood pressure variability in the time domain in patients with recent and long-standing diabetes mellitus. PloS One. 11:e0148378. 10.1371/journal.pone.0148378 26849653 PMC4746070

[B49] Rizzo R. Zhang X. Wang J. W. J. L. Lombardi F. Ivanov P. C. (2020). Network physiology of cortico–muscular interactions. Front. Physiol. 11:558070. 10.3389/fphys.2020.558070 33324233 PMC7726198

[B50] Scholz J. P. Schöner G. (1999). The uncontrolled manifold concept: identifying control variables for a functional task. Exp. Brain Res. 126, 289–306. 10.1007/s002210050738 10382616

[B51] Schulz S. Adochiei F. C. Edu I. R. Schroeder R. Costin H. Bär K. J.. (2013). Cardiovascular and cardiorespiratory coupling analyses: a review. Philos. Trans. R. Soc. A Math. Phys. Eng. Sci. 371:20120191. 10.1098/rsta.2012.0191 23858490

[B52] Seely A. J. E. Macklem P. (2012). Fractal variability: an emergent property of complex dissipative systems. Chaos 22:13108. 10.1063/1.3675622 22462984

[B53] Skinner J. S. McLellan T. H. (1980). The transition from aerobic to anaerobic metabolism. Res. Q. Exerc. Sport 51, 234–248. 10.1080/02701367.1980.10609285 7394286

[B54] Slapsinskaite A. García-Retortillo S. Balagué N. Hristovski R. Tenenbaum G. (2016). Cycling outdoors facilitates external thoughts and endurance. Psychol. Sport Exerc. 27, 78–84. 10.1016/j.psychsport.2016.08.002

[B55] World Health Organization (2000). Ageing and Intellectual Disabilities - Improving Longevity and Promoting Healthy Ageing: Summative Report. Geneva.

[B56] World Medical Association (2013). World Medical Association declaration of Helsinki. JAMA 310, 2191–2194. 10.1001/jama.2013.281053 24141714

[B57] Zago M. Duarte N. A. C. Grecco L. A. C. Condoluci C. Oliveira C. S. Galli M. (2020). Gait and postural control patterns and rehabilitation in Down syndrome: a systematic review. J. Phys. Ther. Sci. 32, 303–314. 10.1589/jpts.32.303 32273655 PMC7113426

[B58] Zebrowska M. Garcia-Retortillo S. Sikorski C. Balagué N. Hristovski R. Javierre C.. (2020). Decreased respiratory coupling in repeated maximal exercise bouts. Europhys. Lett. 132:28001. 10.1209/0295-5075/132/28001

